# The Prevalence of Low Vitamin D in Elite Para-Athletes: A Systematic Review

**DOI:** 10.1186/s40798-024-00756-y

**Published:** 2024-09-04

**Authors:** Christina Kate Langley, Christopher Ian Morse, Aidan John Buffey

**Affiliations:** 1 Department of Sport and Exercise Sciences, Manchester Metropolitan University, Manchester, UK; 2 Manchester Institute of Sport, Manchester Metropolitan University, Manchester, UK; 3 University Academy 92 Ltd, Manchester, UK; 4 Department of Physical Education and Sport Sciences, Faculty of Education and Health Sciences, University of Limerick, Limerick, Ireland; 5 Physical Activity for Health Research Cluster, Health Research Institute, University of Limerick, Limerick, Ireland

**Keywords:** 25(OH)D, Ambulatory, Deficiency, Dietary intake, Disability, Insufficiency, Season, Sport, Wheelchair

## Abstract

**Background:**

Vitamin D insufficiency (25OHD, 50–75 nmolˑl^− 1^) is a common issue within healthy adults and elite athletes and is associated with decreased musculoskeletal health and performance. However, few studies have identified the prevalence and risk factors associated with vitamin D insufficiency within elite Para-Athletes.

**Methods:**

An electronic search was completed on the 5th January 2023 and updated on the 21st June 2024, searching Web of Science, PubMed, Scopus, Cochrane Library and EASY (originally OpenGrey). To meet the eligibility criteria, retrieved studies were required to include at least one baseline measure of a vitamin D biomarker from elite Para-Athletes performing at national or international levels and therefore all quantitative study designs could be included. Risk of bias was assessed using the Joanna Briggs Institute Critical Appraisal Checklist (8-item) for analytical cross-sectional studies. Data from the eligible studies was extracted and charted, with a supporting narrative synthesis.

**Results:**

The search strategy retrieved 3083 articles, of which ten studies met the inclusion criteria. In total there were *n* = 355 Para-Athletes, 69.6% of which comprised of males in the included studies. Across the ten included studies, *n* = 546 samples were taken from *n* = 355 Para-Athletes across different seasons and based upon the 25(OH)D insufficiency and deficiency thresholds set by each individual study 43.2% of the samples were considered insufficient and 28.1% deficient. During the winter months vitamin D insufficiency was at its most prevalent at 74.1%, compared to 57.1% in summer of the 25(OH)D samples measured in Para-Athletes. Wheelchair athletes who competed in indoor sports were also more susceptible to low vitamin D.

**Conclusion:**

This review has highlighted that vitamin D insufficiency and deficiency is highly prevalent in elite level Para-Athletes, all year, across both summer and winter months. Therefore, this review highlights the need for education, treatment, and preventative measures in elite Para-Athletes throughout the year.

**Registration:**

The following systematic review was prospectively registered through PROSPERO International prospective register of systematic reviews (PROSPERO registration ID number: CRD42022362149).

## Key points

74.1% of all 25(OH)D samples taken from elite Para-Athletes were considered insufficient in the winter months, therefore showing a high prevalence rate of low vitamin D.

There is a high prevalence of low vitamin D all year round where 68.1% of samples taken from time points across the year were considered insufficient in elite Para-Athletes.

Based upon group averages indoor elite Para-Athletes from all included studies had low vitamin D all year round.

Dietary vitamin D intake was very poor in all elite Para-Athletes.

Elite Wheelchair Para-Athletes are more likely to be vitamin D deficient based upon group averages compared to elite ambulatory Para-Athletes.

## Background

### Background and Rationale

The role of vitamin D upon human physiological function has been thoroughly researched in recent decades whereby the identification of high levels of deficiency^1^ worldwide continues to drive research in this area [1, 2]. Despite having two major sources of vitamin D through diet and endogenous cutaneous synthesis of ultraviolet beta (UVb) radiation from sun exposure [3] vitamin D deficiency is typically more common than other micronutrient deficiencies. Largely due to dietary vitamin D only accounting for 10–20% of circulating vitamin D as well as numerous risk factors that inhibit vitamin D absorption and metabolism [1, 4]. Risk factors that impact absorption include but are not limited to: sun protection such as clothing and sun cream, outdoor time, skin pigmentation [3] and latitude. Individuals living at latitudes over 35⁰N experience seasonal variations and have severely reduced to negligible amounts of UVb exposure in the winter and autumn months [5]. Likewise, factors such as anti-convulsant medications and kidney diseases can reduce the metabolism of vitamin D [6]. There are also other known modifiable lifestyle factors which impact UVb exposure, for example, sedentary behaviours have been shown to correlate to reductions in outdoor time and sun exposure is likely to be diminished [7]. These risk factors are all likely contributors to the world-wide problem of vitamin D insufficiency and deficiencies [2].

Vitamin D deficiency (< 50 nmolˑl^− 1^ and insufficiency (50–75 nmolˑl^− 1^)) (hereby known as ‘low vitamin D’) is known to reduce regulation of intestinal calcium absorption which is a major determinant of calcium homeostasis [8]. As vitamin D plays a central role in calcium absorption, individuals with low vitamin D are most commonly associated with diminished bone health [9]. To address the deterioration of bone health, an abundance of research has focused on ageing populations who are susceptible to low bone density and supplementing vitamin D as an intervention to reduce fractures and osteoporosis risk [10–13]. There is however growing evidence around the impact of vitamin D insufficiency and deficiency upon other non-skeletal systems such as, reductions in immune function [14] and skeletal muscle strength and function [15–17]. A systematic review and meta-analysis identified six randomised control trials that assessed the impact of vitamin D supplementation on muscle strength in young adults compared to controls and identified significant increases in upper and lower limb strength after supplementation [18]. With this growing acknowledgement that vitamin D deficiency does indeed impact muscle strength and bone health, research into vitamin D has shifted over to athletic populations to identify the impact of vitamin D insufficiency and deficiency on decrements in performance, recovery rate as well as its impact on injuries and illness in athletes [19–21]. Early research, informed by the detrimental effects of low vitamin D, investigated the effect of supplementation beyond sufficient to an ‘optimal’ level to identify if vitamin D could indeed improve performance, in athletes [20]. However, the benefits of shifting vitamin D levels from ‘sufficient’ to ‘optimal’ were negligible [20], whereas previous research has shown that musculoskeletal performance only improves in individuals who had low vitamin D as a baseline [22]. These findings justify the recommendation and need for athletes to have sufficient levels of vitamin D, to mitigate reductions in normal physiological function [22]. However, numerous studies have identified that the high prevalence of low vitamin D that is reported across general non-athletic populations is just as prominent in athletic populations and is therefore accompanied by decrements in musculoskeletal health and performance [19, 23–26]. The prevalence of low vitamin D has been shown to differ when comparing the type of sport played, for example, Constatini et al. [27] showed indoor athletes to be at an elevated risk of vitamin D insufficiency compared with outdoor athletes, with 80% compared to 48% being insufficient, respectively. The risk of vitamin D insufficiency and deficiency in athletes has been shown at varying latitudes and unsurprisingly a study based in the United Kingdom (UK) (53⁰N) showed insufficient levels of vitamin D < 75 nmolˑl^− 1^ in all athletes assessed from rugby players, footballers, flat and jump jockeys during the winter [28]. Although the risks of insufficiency are higher in less equatorial populations, athletes living in countries close to the equator where vitamin D would typically be assumed to be sufficient, have also been shown to be at risk as illustrated in an Australian study (35⁰S) by Lovell [29] who found insufficiency in 83% of their female gymnasts. The importance of vitamin D sufficiency (> 75 nmolˑl^− 1^) in athletic performance is highlighted by numerous literature reviews that show improvements in lower limb muscle strength and athletic performance when insufficient athletes increase their circulating 25(OH)D levels to sufficient levels [30–32]. With the known benefits that sufficient levels of vitamin D have on musculoskeletal performance, injury reduction and general health in both athletic and clinical populations [30, 33], it is surprising that there is a dearth of literature on the prevalence and treatment of low vitamin D in athletes with disabilities (hereby known as Para-Athletes).

Para-Athletes may encompass problems that both athletic and clinical populations experience due to low vitamin D. A major issue that many Para-Athletes face is a predisposition to reduced musculoskeletal health and higher risk of injury compared to that of typically developed athletes [34]. Site-specific reductions in muscle and bone health are often due to secondary implications caused by decreased musculoskeletal loading resulting from reduced levels of ambulation and range of movement in individuals with physical disabilities such as cerebral palsy (CP), spina bifida and SCI [35–37]. Despite Para-Athletes typically showing higher levels of physical activity than sedentary non-athletes with disability [38], Para-Athletes with neurological and musculoskeletal impairments may experience lower muscle strength and bone health compared to typically developed, age matched controls, such as ambulatory athletes with CP. For example, Langley et al. [34] found that Para-Athletes with CP had a 23.7% smaller vastus lateralis CSA, a 40.5% smaller knee extensor strength and a radius T score that was − 1.75 standard deviations (SDs) less compared to typically developed controls [34]. These loses in bone health are even more prominent in wheelchair (WC) based Para-Athletes where osteopenia and osteoporosis have been identified in 45.8% and 12.5% of WC based athletes with SCI [39]. The musculoskeletal health of Para-Athletes may be exacerbated by low vitamin D due to numerous risk factors that reduce vitamin D absorption and metabolism such as sedentary behaviour and medications. Therefore, it is important to identify the prevalence of low vitamin D in Para-Athletes and the associated risk factors (both non-modifiable and modifiable) to help inform future research. Furthermore, the findings may help to inform future interventions that aim to improve the musculoskeletal health, performance and injury risk in Para-Athletes.

### Aims

This present systematic review aimed to (1) identify the prevalence of vitamin D insufficiency in national to elite level Para-Athletes, and (2) identify risk factors which account towards vitamin D insufficiency in elite Para-Athletes.

### Research Questions

(1) What is the prevalence of vitamin D insufficiency in national to elite Para-Athletes worldwide? (2) What are the risk factors which is associated with vitamin D insufficiency in elite Para-Athletes?

## Methods

### Data Sources and Search Strategy

This systematic review was conducted according to the PRISMA guidelines (please see Supplementary File 1 for completed PRISMA Checklist) [40]. A protocol for this review was prospectively registered with PROSPERO (registration ID number: CRD42022362149) prior to data extraction. The PECO framework was used a priori to inform the development of the search strategy:

(P) Population: Elite level Para-Athletes playing at a national level or above. (E) Exposures: Latitude, season, diet, ethnicity, age, descriptive characteristics, physical activity level, outdoor exposures, direct sun exposures, sun protection used, ambulation levels, season, impairment type, type of sport and vitamin D assay used. (C) Comparison: We did not wish to compare interventions or treatments (this is typical in some PECO analysis frameworks, where a comparison is not always present). (O) Outcome: Serum total 25(OH)D levels and prevalence of vitamin D insufficiency and deficiency.

Two independent investigators (C.K.L. and A.J.B.) conducted the systematic literature search, retrieving articles published prior to the 5th January 2023 to the earliest published record and updated searches were completed on the 25th September 2023 and on the 21st June 2024. The electronic search was completed across five databases: Web of Science Core Collection, PubMed, Scopus, Cochrane Library and EASY (originally OpenGrey) using a search strategy that was developed in conjunction with a University Librarian (See Supplementary File 2 for full search strings used for each database, including MEsH terms). The reference lists of the included studies were manually searched.

### Eligibility Criteria and Paper Selection

Inclusion criteria were: (1) English language publication; (2) participants aged ≥ 16 years; (3) participants were Para-Athletes playing at national level or above; (4) assessed for ≥ one biomarker of vitamin D at un-supplemented levels. Studies were excluded if: (1) participants were impacted by diseases which impact the metabolism and/or synthesis of vitamin D; (2) participants were supplemented prior to study commencement, without providing baseline measures. These criteria allowed for direct evaluation between impairment type, ambulation levels, type of sport (indoor or outdoor), latitude and diet.

Retrieved articles were reviewed by two reviewers independently (C.K.L. and A.J.B.); the retrieved studies were screened firstly, by title and abstract based upon the inclusion criteria, and then the full text to confirm eligibility. Any disparity regarding eligibility was discussed between the two independent reviewers until a consensus was reached (Fig. 1).

### Quality Assessment

The Joanna Briggs Institute (JBI) Critical Appraisal Checklist (8-item) for analytical cross-sectional studies was used to assess the risk of bias (quality) of the included studies within this observational systematic review. This quality assessment was completed by separate and blinded independent reviewers (C.K.L. and A.J.B.) who were blinded to each-other. Any disagreements between reviewers were discussed until a consensus was made. The questions (see below) were scored quantitatively out of 8.

Questions included: (1) Were the criteria for inclusion in the sample clearly defined? (2) Were the study subjects and the setting described in detail? (3) Was the exposure measured in a valid and reliable way? (4) Were objective, standard criteria used for measurement of the condition? (5) Were confounding factors identified? (6) Were strategies to deal with confounding factors stated? (7) Were the outcomes measured in a valid and reliable way? (8) Was appropriate statistical analysis used?

### Definition of Low Vitamin D

In this review the definitions of vitamin D deficiency and insufficiency were combined and referred to as ‘low vitamin D’. All studies reported 25(OH)D the major circulating metabolite of vitamin D, but the 25(OH)D measures were either reported as nmolˑl^− 1^ or ngˑml^−1^. For this systematic review, we converted and reported all 25(OH)D measures in nmolˑl^− 1^ by multiplying 25(OH)D in ngˑml^− 1^ by 2.496 (1 ngˑml^− 1^ = 2.496 nmolˑl^−1^).

### Data Extraction and Synthesis

Microsoft Excel sheets (Microsoft Excel, Version 2212) were developed and confirmed by the research team and used for the extraction of data. Outcomes extracted in the review were, study characteristics, participant characteristics, impairment type, sport played, vitamin D outcome measures including dietary vitamin D intake, vitamin D metabolite measured, vitamin D levels, latitude and the season that vitamin D was collected in, from each article which was manually extracted by the first author (C.K.L.) from the included studies. Due to the heterogeneity in study design of the included studies, undertaking a meta-analysis was deemed not to be appropriate. The analysis of the eligible retrieved studies is presented via a narrative synthesis, reporting, and critically appraising the study design, target populations, setting, and outcomes of interest that relate to vitamin D. The descriptive information and variables reported from the included studies were extracted and included within this systematic review (See Tables 1 and 2).

## Results

### Literature Search

**Fig. 1:**
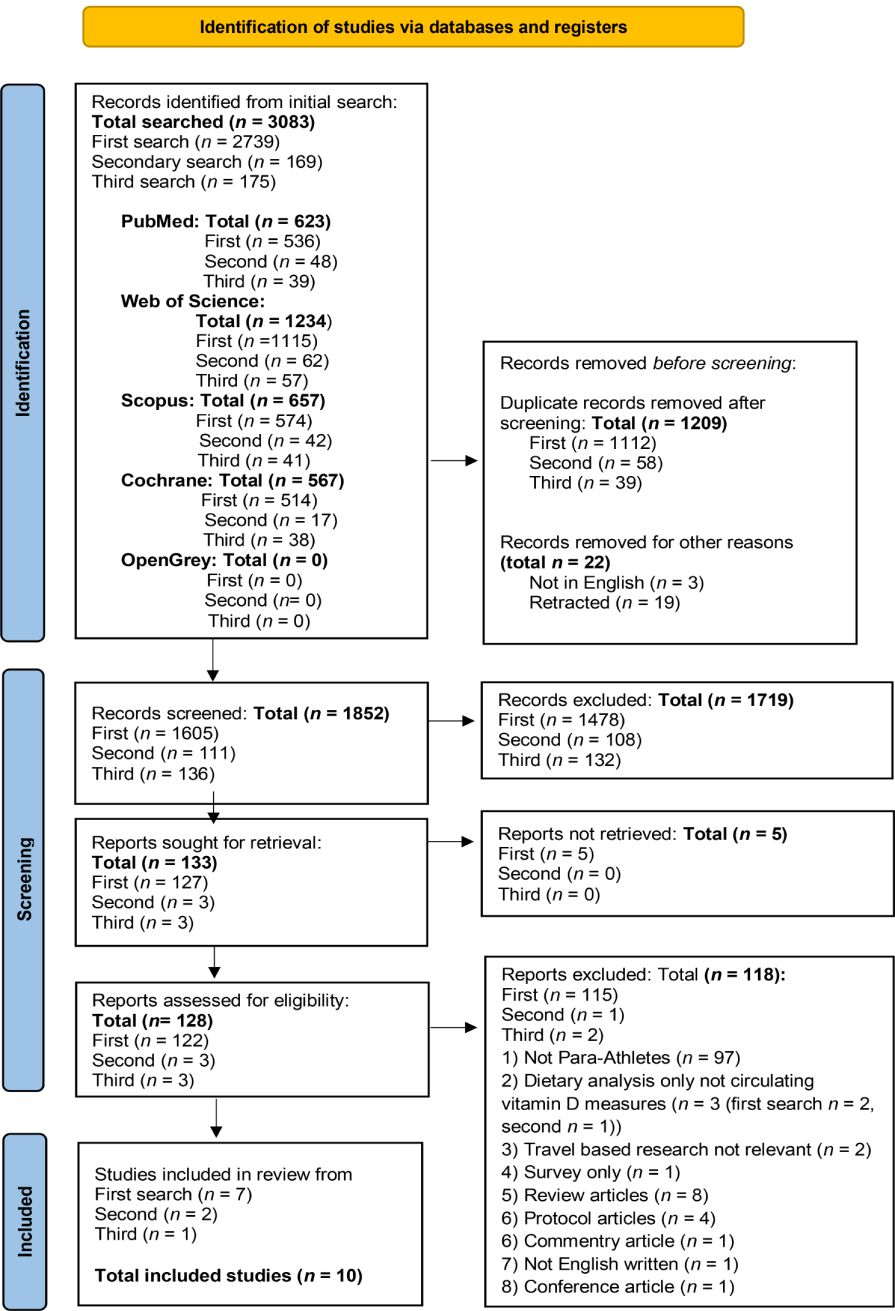
The PRISMA flow diagram illustrating the number of studies retrieved and how many were assessed for eligibility before being excluded with reasons, leaving the final *n* = 10 of included studies, following the PRISMA 2020 statement: an updated guideline for reporting systematic reviews [40]

### Quality Assessment and Risk of Bias

The quality assessment scores of each article are reported in Table 1. The quality of seven of the ten included studies was mutually agreed upon, with three of the studies included requiring discussion until a consensus was met.

### Study Characteristics and Descriptors

Table 1 provides details of the study characteristics of the ten included articles. Of the ten included studies, three of the studies were completed in Switzerland, two in the United Kingdom (UK), two across the USA and Canada, one in Ireland, one in Norway and one in Lithuania. All studies included were performed at latitudes above the equator between 37–62°N and therefore experienced seasonal variations. Two studies described one season of 25(OH)D in Para-Athletes, five studies collected repeated measures of 25(OH)D throughout the year, three studies collected pre and post vitamin D supplementation whereby for the purpose of this review only baseline pre-supplemented measures were extracted [41] (Table 1).

**Table 1:** Included study information

First Author	Publication Year	Origin of Study	Study Design	Seasons	Sample Size (*n*)	Sex	Latitude	Vitamin D Measure	Quality Score (/8)
Magee et al.	2013	Ireland	Observational study	Winter	33	M = 24 (72.7%) F = 9 (27.3%)	53°N	ELISA; IDS Octeia 25-hydroxy vitamin D enzyme immunoassay [Immunodiagnostic Systems Limited]	4
Flueck et al.	2016a	Switzerland	Retrospective analysis	Winter, Summer	72	M = 51 (70.8%) F = 21 (29.2%)	47°N	Venous and Enzyme linked fluorescent assay tech (VIDAS)	6
Flueck et al.	2016b	Switzerland	Double blind, non-randomised intervention study	Winter	21	M = 21 (100%)	47°N	Venous and Enzyme linked fluorescent assay tech (VIDAS)	8
Pritchett et al.	2016	USA and Canada	Longitudinal, observational trial	Autumn, Winter	39	M = 19 (48.7%) F = 20 (51.3%)	37–56°N	Blood spots and blood spot assay	8
Pritchett et al.	2017	USA and Canada	Longitudinal interventional study	Winter	35	M = 30 (85.7%) F = 5 (14.3%)	37–56°N	Blood spots and blood spot assay	6
Baranauskas et al.	2020	Lithuania	Observational cross-sectional study	Summer	14	F = 14 (100%)	55°N	Haematological analyser MEK6400-K	7
Langley et al.	2021	UK	Cross-sectional	Winter	24	M = 24 (100%)	53°N	Enzyme linked immunosorbent assay (ELISA) Orgentec	8
Langley et al.	2023	UK	Longitudinal, Cross-sectional study	Winter, Summer	16	M = 16 (100%)	53°N	Enzyme linked immunosorbent assay (ELISA) Orgentec	7
Hertig-Godeschalk et al.	2023	Switzerland	Randomised controlled crossover trial	Unidentifiable (March-Oct)	14	M = 6 (57.2%) F = 8 (43.8%)	47°N	Electrochemiluminescence immunoassay (ECLIA), Cobas 6000 e601	5
Steffen et al.	2024	Norway	Longitudinal observation study	Winter, Spring, Summer, Autumn	87	M = 56 (64%) F = 31 (36%)	62°N	-	4

*Abbreviations* UK – United Kingdom, USA – United States of America. M = Male, F = Female and N = North

### Participant Characteristics

In total, 355 participants were sampled in the ten included studies. All ten of the included studies used elite Para-Athletes performing at national or international level (see Table 2 which illustrates impairment types and sports played). Nine of the included studies included male participants (total *n* = 247, 69.6%), whereas seven studies included female participants (total *n* = 108, 30.4%). Age was collected in all ten studies. Standing stature was measured in seven of ten studies. One study collected seated stature of their sampled WC Para-Athletes [42]. Eight of the ten studies collected body mass. Two studies did not report stature or mass of their participants [43, 44].

**Table 2:** Participant descriptive characteristics presented, including impairment type, anthropometric measures, ethnicity, ambulation levels, sport played and level of performance, playing environment and physical activity levels

Article First Author	Para-Athlete Impairment Type	Sport(s)	Age (years)	Stature (cm)	Body Mass (kg)	Ethnicity	Ambulation Levels	Level of Performance	Indoor or Outdoor Sport	Physical Activity (PA) and Training Data
Magee et al. 2013	Paralympians	Athletics, Football, Boccia, Rowing	32 (22, 39) (median and range)	-	-	-	WC *n* = 12 Ambulatory *n* = 21	Elite	-	-
Flueck et al. 2016a	Paraplegic, Tetraplegic, Amputees	Basketball, Rugby, Table Tennis, Alpine Skiing	M = 34 ± 13 F = 31 ± 13	M = 174 ± 10 F = 162 ± 12	M = 70 ± 13 F = 56 ± 13	-	WC *n* = 72	Elite	Both – Indoor and Outdoor	-
Flueck et al. 2016b	SCI CP	Basketball, Rugby, Table Tennis	36 ± 12	180 ± 8	72 ± 15	-	WC *n* = 21	Elite	Indoor	6.3 ± 3.7 h/week
Pritchett et al. 2016	SCI, SB	Tennis, Athletics, Basketball, Rugby	27.7 ± 6.5	131.5 ± 13.6 (seated assumed)	59.5 ± 13.5	African American *n* = 1 White Hispanic *n* = 1 Asian *n* = 3 Caucasian *n* = 30	WC *n* = 39	Elite	Both (*n* = 15 outdoor and 24 indoor)	-
Pritchett et al. 2017	SCI, SB, Cauda Equina	Athletics, Rugby	33 ± 15	170.2 ± 25.4	69.6 ± 28.2	Asian *n* = 4, African American *n* = 1, White (Caucasian) *n* = 30	WC *n* = 35	Elite (Paralympians or national)	-	-
Baranauskas et al. 2020	Deaf	Basketball	26.4 ± 4.5	171 ± 6.2	65.2 ± 7.8	-	Ambulatory *n* = 14	Elite (Deaflympians)	Indoor *n* = 14	PA frequency /week 4–6 PA duration (mins/session) 52.2 ± 25.2 PA total (mins/week) 359.1 ± 173.0
Langley et al. 2021	CP	Football	21.0 ± 1.4	174 ± 7	66.4 ± 10.1	-	Ambulatory *n* = 24	Elite	Outdoor	PA frequency /week – 4.00 ± 1.84 PA duration (mins/session) 65.2 ± 28.3 PA total (mins/week) 251.3 ± 135.0 Step count 8218 ± 3292
Langley et al. 2023	CP	Football	21 ± 1.3	176 ± 6	68.8 ± 6.7	-	Ambulatory *n* = 16	Elite	Outdoor	IPAQ 8737 ± 3975 PA frequency/ week 4.0 ± 1.9 PA duration (mins/session) 70.1 ± 32.2 Step count 10,696 ± 3987
Hertig-Godeschalk et al. 2023	SCI = 6 Meningomyelocele = 5 MS = 2 Arthrogryposis = 1	Handcycling = 4 NS = 10	M = 36 ± 8 F = 32 ± 11	M = 172 ± 7 F = 159 ± 14	M = 58 ± 7 F = 59 ± 12	-	WC *n* = 14	Elite	Outdoor *n* = 11 Indoor *n* = 3	PA duration 14 ± 5 h/week
Steffen et al. 2024	Neurological = 51 Musculoskeletal = 32 Visual = 4	Summer sports = 39 Winter sports = 48	31.7 (16–62)	-	-	-	-	Elite	-	PA duration 14 ± 5 h/week
*Abbreviations* M-Male, F-Female, SCI – Spinal cord injury, SB-Spina Bifida, C-Cervical, T-Thoracic, L-Lumbar, CP-Cerebral Palsy, WC-Wheelchair, NS = Not Specified, PA-Physical Activity, IPAQ – International Physical Activity Questionnaire. - denotes unreported in study.										

### Dietary Vitamin D

Five of the ten studies reported dietary vitamin D, of which all five measured dietary intakes in the winter months [34, 42, 43, 45, 46] and just one of those five reported dietary vitamin D in the autumn [46]. All of the five studies, which reported dietary intake, were well below the Institute of Medicines (IOM) recommended daily intake (RDI) of 400–600 international units (IU) [47, 48]. Baranauskas et al. [45] reported an average vitamin D intake of 244 ± 228 IU/d (61% of RDI). Langley et al. [34] found that the average dietary vitamin D intake was 166 ± 186 IU/d (41.5% of RDI). Pritchett et al. [46] reported Para-Athletes to have a dietary intake of 115 ± 12.25 IU/d in the winter (29.75% of RDI). Para-Athletes in Pritchett et al. [42] averaged 212 ± 103 IU/d (53% of RDI). Magee et al. [43] reported a daily vitamin D intake of 5.49 µg/d (219 IU/d) (54.75% of RDI). Pritchett et al. [46] was the only study that reported autumn dietary vitamin D in Para-Athletes which was well below the RDI at 121.1 ± 9.8 IU/d (30.25% of RDI). No dietary vitamin D was reported in the summer or spring months. Three of the five studies analysed the relationship between dietary vitamin D and 25(OH)D and all reported no association of dietary vitamin D with 25(OH)D in Para-Athletes [34, 43, 46].

### Measurement of Vitamin D

All ten of the included studies measured the vitamin D metabolite 25(OH)D, measured via different techniques. Seven studies collected venous blood samples of those seven, three of the included studies performed enzyme linked Immuno-Sorbent Assays [34, 43, 49], two studies performed enzyme linked florescent assays [41, 50], one study used electrochemiluminescence immunoassay (ECLIA) [51] and one study analysed their samples using a haematological analyser [45]. Two studies performed fingertip blood spots and blood spot assays [42, 46] (Table 1). Six studies collected 25(OH)D samples from all participants except for two studies that reported participants who did not provide a 25(OH)D sample due to fear of needles with *n* = 2 from Langley et al. [34] and *n* = 1 from Langley et al. [49] not providing samples. One study did not identify the method of 25(OH)D assessment [44].

### Vitamin D Status Thresholds

Eight of the ten studies used the threshold for vitamin D sufficiency as > 75 nmolˑl^− 1^ (or 30 ngˑml^− 1^), with their insufficiency thresholds being between 75 − 50 nmolˑl^− 1^ (30 − 20 ngˑml^− 1^) and the deficiency thresholds being set at < 50 nmolˑl^− 1^ (< 20 ngˑml^− 1^) (Table 3). The study by Pritchett et al. [46] used a sufficiency threshold of < 80 nmolˑl^− 1^ (< 32 ngˑml^− 1^), with their insufficiency thresholds being between 80 − 50 nmolּˑl^− 1^ (32 − 20 ngˑml^−1^) and the deficiency thresholds being the same as the six studies above set at < 50 nmolˑl^− 1^ (< 20 ngˑml^− 1^). The study by Magee et al. [44] used a lower sufficiency threshold of > 50 nmolˑl^− 1^ (> 20 ngˑml^− 1^) and used a threshold of < 50 nmolˑl^− 1^ (< 20 ngˑml^− 1^) for insufficiency but did not state one for deficiency (See Table 3). Four of the ten studies also included severely deficient thresholds, where two studies set a threshold at < 27.5 nmolˑl^− 1^ (< 13 ngˑml^− 1^) [42, 51] and two at < 25 nmolˑl^− 1^ (< 12 ngˑml^− 1^) [35, 50] (Table 3).

**Table 3:** 25(OH)D outcomes extracted from included articles

Article	Insufficiency Thresholds Used in Article	Season(s) / Time Point(s) 25(OH)D Measured	Samples Insufficient or Below	Samples Sufficient	Samples Insufficient	Samples Deficient	Samples Severely Deficient	Serum 25(OH)D Levels (Total Samples)
Magee et al. 2013	25(OH)D Insufficiency < 50 nmolˑl^− 1^	Winter (Nov) 2010)	*n* = 9 (27%)	*n* = 24 (73%)	*n* = 9 (27%)	-	-	57.9 (48.9–67.7) nmolˑl^− 1^ 23.3 (19.6–27.2) ngˑml^− 1^
Flueck et al. 2016a	25(OH)D Insufficiency < 75 nmolˑl^− 1^ Deficiency < 50 nmolˑl^− 1^ Severe deficiency as < 27.5 nmolˑl^− 1^	Total across whole year	*n* = 120 (73.2%)	*n* = 44 (26.8%) 89.8 ± 12.6 nmolˑl^− 1^ 36.1 ± 5.1 ngˑml^− 1^	*n* = 61 (37.2%) 62.0 ± 6.4 nmolˑl^− 1^ 24.8 ± 2.6 ngˑml^− 1^	*n* = 47 (28.7%) 38.7 ± 6.3 nmolˑl^− 1^ 15.5 ± 2.5 ngˑml^− 1^	*n* = 12 (7.3%) 20.6 ± 4.5 nmolˑl^− 1^ 8.3 ± 1.8 ngˑml^− 1^	-
		Summer (May-Oct)	*n* = 48 (61.5%)	*n* = 30 (38.5%)	*n* = 34 (43.6%)	*n* = 13 (16.7%)	*n* = 1 (1.3%)	69.5 ± 21.4 nmolˑl^− 1^ 27.9 ± 8.6 ngˑml^− 1^ 32–119 nmolˑl^− 1^ 13–48 ngˑml^− 1^
		Winter (Nov- April)	*n* = 72 (83.7%)	*n* = 14 (16.3%)	*n* = 27 (31.4%)	*n* = 34 (39.4%)	*n* = 11 (12.8%)	51.5 ± 21.9 nmolˑl^− 1^ 20.7 ± 8.8 ngˑml^− 1^ 13–109 nmolˑl^− 1^ 5–44 ngˑml^− 1^
Flueck et al. 2016b	25(OH)D Insufficiency < 75 nmolˑl^− 1^ Deficiency < 50 nmolˑl^− 1^ Severe deficiency < 27.5 nmolˑl^− 1^	Winter (Nov-April)	*n* = 20 (100%)	-	-	-	-	44 ± 18 nmolˑl^− 1^ 17.7 ± 7.2 ngˑml^− 1^
Pritchett et al. 2016	25(OH)D Insufficiency < 80 nmolˑl^− 1^ Deficiency < 50 nmolˑl^− 1^	Autumn (October)	*n* = 26 (66.7%)	*n* = 13 (33.3%)	*n* = 20 (51.3%)	*n* = 6 (15.4%)	-	69.6 ± 19.7 nmolˑl^− 1^ 28.0 ± 7.9 ngˑml^− 1^ 30–107 nmolˑl^− 1^ 12–43 ngˑml^− 1^
		Winter (Feb/Mar)	*n* = 18 (56.4%)	*n* = 14 (43.6%)	*n* = 13 (41%)	*n* = 5 (15.4%)	-	67.4 ± 25.5 nmolˑl^− 1^ 27.1 ± 10.2 ngˑml^− 1^ 20–117 nmol l^− 1^ 8–47 ngˑml^− 1^
Pritchett et al. 2017	25(OH)D Insufficiency < 75 nmolˑl^− 1^ Deficiency < 50 nmolˑl^− 1^	Winter (Nov-April)	*n* = 25 (74%)	*n* = 9 (26%) 98.0 ± 19.3 nmolˑl^− 1^ 39.4 ± 7.8 ngˑml^− 1^	*n* = 17 (50%) 62.8 ± 8.3 nmolˑl^− 1^ 25.2 ± 3.3 ngˑml^− 1^	*n* = 8 (24%) 38.8 ± 6.0 nmolˑl^− 1^ 15.6 ± 2.4 ngˑml^− 1^	-	66.3 ± 24.3 nmolˑl^− 1^ 26.6 ± 9.8 ngˑml^− 1^
Baranauskas et al. 2020	25(OH)D Insufficiency 75 − 50 nmolˑl^− 1^ Deficiency < 50 nmolˑl^− 1^	Summer (June)	*n* = 12 (85.7%)	*n* = 2 (14.3%)	*n* = 8 (57.1%)	*n* = 4 (28.6%)	-	60.0 ± 16.4 nmolˑl^− 1^ 24.1 ± 6.6 ngˑml^− 1^
Langley et al. 2021	25(OH)D Insufficiency 75 − 50 nmolˑl^− 1^ Deficiency 49.9–30 nmolˑl^− 1^ Severe deficiency <30nmol l^− 1^	Winter (Feb-Mar)	*n* = 20 (90.9%)	*n* = 2 (9.1%)	*n* = 8 (36.4%)	*n* = 7 (31.8%)	*n* = 5 (22.7%)	46.6 ± 18.2 nmolˑl^− 1^ 18.7 ± 7.3 ngˑml^− 1^
Langley et al. 2023	25(OH)D Insufficiency *(*75 − 50 nmolˑl^− 1^	Winter (Feb-Mar)	*n* = 11 (73.3%)	*n* = 4 (26.7%)	*n* = 6 (40%)	*n* = 5 (33.3%)	-	40.1 ± 15.7 nmolˑl^− 1^ 16.1 ± 6.3 ngˑml^− 1^
	Deficiency 49.9–30 nmolˑl^− 1^ Severe deficiency <30 nmolˑl^− 1^	Summer (May-Sept)	*n* = 3 (20%)	*n* = 12 (80%)	*n* = 3 (20%)	*n* = 0 (0%)	-	76.7 ± 31.1 nmolˑl^− 1^ 30.8 ± 12.5 ngˑml^− 1^
Hertig-Godeschalk et al. 2023	25(OH)D Insufficiency < 75 nmolˑl^− 1^	Total	*n* = 11 (78.6%)	*n* = 3 (21.4%)	-	-	-	72 ± 17 nmolˑl^− 1^ 28.9 ± 6.8 ngˑml^− 1^
		T1–4 weeks	*n* = 9 (64.3%)	*n* = 5 (35.7%)	-	-	-	-
		T2–8 weeks	*n* = 6 (42.9%)	*n* = 8 (57.1%)	-	-	-	-
		T3–12 weeks	*n* = 7 (50%)	*n* = 7 (50%)	-	-	-	-
Steffen et al. 2024	25(OH)D Insufficiency < 75 nmolˑl^− 1^	Total	*n* = 75 (74%)	*n =* 12 (26%)	-	-	-	61.0 ± 30.0 nmolˑl^− 1^ 24.5 ± 12.0 ngˑml^− 1^ 18–216 nmolˑl^− 1^ 7.2–86.7 ngˑml^− 1^
	Deficiency < 50 nmolˑl^− 1^	Winter	*n =* 28 (88%)	*n =* 4 (12%)	*n =* 8 (25%)	*n* = 16 (53%)	-	50.0 ± 18.0 nmolˑl^− 1^ 20.1 ± 7.2 ngˑml^− 1^ 20–90 nmolˑl^− 1^ 8–36 ngˑml^− 1^
		Spring	*n =* 25 (63%)	*n =* 14 (37%)	*n =* 14 (35%)	*n =* 11 (28%)	-	69.0 ± 37.0 nmolˑl^− 1^ 27.7 ± 14.9 ngˑml^− 1^ 18–216 nmolˑl^− 1^ 7.2–86.7 ngˑml^− 1^
		Summer	*n* = 1 (25%)	*n* = 4 (75%)	*n =* 1 (25%)	*n =* 0 (0%)	-	101.0 ± 38.0 nmolˑl^− 1^ 40.6 ± 15.3 ngˑml^− 1^ 27–66 nmolˑl^− 1^ 11–27 ngˑml^− 1^
		Autumn	*n =* 21 (81%)	*n =* 5 (19%)	*n =* 11 (43%)	*n =* 10 (38%)	-	60.0 ± 21.0 nmolˑl^− 1^ 24.1 ± 8.4 ngˑml^− 1^ 32–123 nmolˑl^− 1^ 13–49 ngˑml^− 1^
		Totals	*n =* 372 (68.1%)	*n =* 174 (31.9%)	*n =* 236 (43.2%)	*n =* 119 (21.8%)	*n =* 17 (3.1%)	62.6 ± 22.9 nmolˑl^− 1^ 25.2 ± 9.2 ngˑml^− 1^
		Winter	*n =* 203 (74.1%)	*n =* 71 (25.9%)	*n =* 112 (40.9%)	*n =* 75 (27.4%)	*n =* 16 (5.8%)	53 ± 20.2 nmolˑl^− 1^ 21.3 ± 8.1 ngˑml^− 1^
		Spring	*n =* 25 (64.1%)	*n =* 14 (35.9%)	*n =* 14 (35.9%)	*n =* 11 (28.2%)	-	69.0 ± 37.0 nmolˑl^− 1^ 27.7 ± 14.9 ngˑml^− 1^
		Summer	*n =* 64 (57.1%)	*n =* 48 (42.9%)	*n =* 46 (41.1%)	*n =* 17 (15.2%)	*n =* 1 (1%)	31.9 ± 19.3 nmolˑl^− 1^ 18.3 ± 8.6 ngˑml^− 1^
		Autumn	*n =* 47 (72.3%)	*n =* 18 (27.7%)	*n =* 31 (47.7%)	*n =* 16 (24.6%)	-	31.3 ± 18.5 nmolˑl^− 1^ 18.0 ± 7.9 ngˑml^− 1^
		Not Specified	*n =* 33 (58.9%)	*n =* 23 (41.1%)	*n =* 33 (58.9%)	-	-	32.2 ± 17.6 nmolˑl^− 1^ 17.9 ± 6.8 ngˑml^− 1^

*Abbreviations* 25(OH)D − 25-Hydroxyvitamin D

### Seasons Measured and Low 25(OH)D Prevalence

In total the ten included studies collected a total of *n* = 546 25(OH)D samples from the *n* = 355 participants in all studies combined (Table 3). Of the ten studies included, one study collected multiple 25(OH)D samples over a 12-month period overall totalling *n* = 154 samples from *n* = 72 participants. Eight studies reported 25(OH)D in the winter months which included *n* = 274 samples from a total of *n* = 250 participants. One study collected 25(OH)D *n =* 39 samples in the spring from *n* = 39 participants [44]. Four studies collected a total of *n* = 112 25(OH)D samples in the summer months in a total of *n* = 107 participants [44, 45, 49, 50]. Two studies collected *n* = 65 25(OH)D samples in the autumn from *n* = 65 participants [42, 44]. One study collected 25(OH)D samples between March and October, but 25(OH)D samples were separated into 4 different time points over a 12-week period based upon the Para-Athletes training schedule (T0-baseline, T1-4 weeks, T2-8 weeks, T3–12 weeks) meaning that seasonal means were not identifiable [51].

**Fig. 2:**
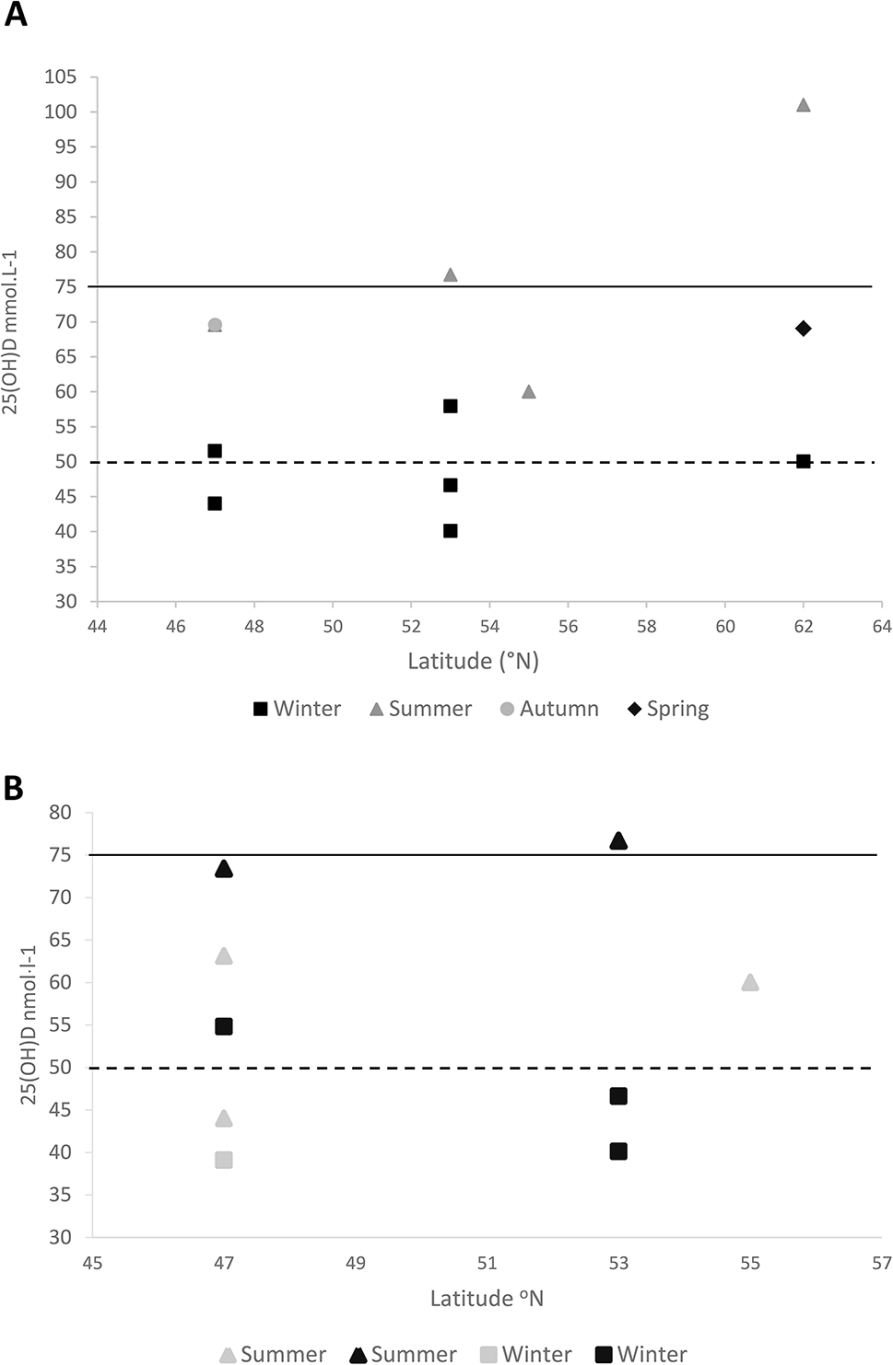
**A** & **B**. Displays the reported 25(OH)D levels in Para-Athletes plotted against the latitude (°N) that participants resided in each study. The legends identify the descriptives being compared. Solid lines denote threshold for vitamin D insufficiency (< 75 − 50 nmolˑl^− 1^) and dashed lines denote threshold for vitamin D deficiency (< 50 nmolˑl^− 1^). Two studies were not included in these figures due to them providing a latitude range between 37–56^°^N and therefore could not be plotted [42, 46]. One study was not included due to 25(OH)D being averaged from samples taken across different seasons making it unidentifiable [51]

Based upon the insufficiency thresholds used for this systematic review (insufficient 75 − 50 nmolˑl^− 1^, deficient 49.9–30 nmolˑl^− 1^, severely deficient (< 30 nmolˑl^− 1^) in the winter months there was low 25(OH)D on average in all Para-Athletes based on the eight studies that measured 25(OH)D in the winter. Where four studies reported average levels that were insufficient [42, 43, 46, 50], and four studies reported levels that were considered deficient [34, 41, 44, 49]. In the summer months 25(OH)D was reported in Para-Athletes by four studies, where two studies reported an average 25(OH)D that was insufficient [45, 50] and two studies showed 25(OH)D to be sufficient in the summer months [44, 49]. Two studies measured 25(OH)D in Para-Athletes in the autumn which were both on average insufficient [44, 46]. One study that took mean 25(OH)D between March and October reported insufficient levels of 25(OH)D [51]. One study presented mean 25(OH)D in the spring months which was considered insufficient [44](See Fig. 2A). Two studies investigated seasonal variations, whereby Langley et al. [49] found a 70.5% increase *p* = 0.003 from winter (deficient on average) to summer months (insufficient on average). Whereas Pritchett et al. [46] found that there were no significant differences between autumn or winter months in their Para-Athletes.

### Sex and Vitamin D

Six studies collected 25(OH)D samples from both male and female Para-Athletes [42–44, 46, 50, 51], four of which did not separate male and female samples, therefore these four studies were not able to be compared by sex [42–44, 51]. One study collected from females only [45] and three collected from males only [34, 42, 49]. Of the three studies that measured female Para-Athletes, two studies collected 25(OH)D over two seasons, winter and summer [50] and winter and autumn [46] and one study collected 25(OH)D during the summer only [45]. All included studies that solely measured or separated female Para-Athletes, illustrated that they were insufficient in 25(OH)D despite measuring in different seasons [43, 45, 46]. Of the six studies that measured male Para-Athletes, three studies collected 25(OH)D over two seasons, winter and summer [49, 50] and winter and autumn [46] and two studies collected from males only in the winter months [34, 41]. Of those five studies that measured males in the winter months four showed deficiency, [34, 41, 49, 50] and one showed insufficiency [46]. Whereas, when investigating the studies that measured within the summer period, one study showed male Para-Athletes were insufficient [50] and one study found male Para-Athletes to be sufficient in the summer [49]. In the one study that measured 25(OH)D in the autumn, male Para-Athletes were classified as insufficient on average [46]. Two of the included studies compared differences between sex and 25(OH)D within their own study [46, 50]. Whereby Flueck et al. [50] showed no significant difference between sex across the whole year and Pritchett et al. [46] concluded that 25(OH)D was not significantly different between sex in the autumn (*p* = 0.29) or winter (*p* = 0.59).

### Training Environment on 25(OH)D

Two of the included studies measured 25(OH)D and reported separately indoor and outdoor training Para-Athletes within the published manuscript [46, 50]. Whereas two of the included studies sampled and reported outdoor Para-Athletes only [34, 49] and two of the included studies included and reported on 25(OH)D for indoor Para-Athletes only [41, 45] (Table 4). Four studies did not report the training environments of their Para-Athletes [42–44, 51].

**Table 4:** 25(OH)D samples across differences across sex, sports environment and ambulation level during each season

		25(OH)D across Sexes		25(OH)D across Sports Environments		Serum 25(OH)D based on Ambulation Level	
Article	**Season**	**Male**	**Female**	**Indoor Athletes**	**Outdoor** **Athlete**	**Wheelchair**	**Ambulatory**
Magee et al. 2013	Winter	-	-	-	-	*n* = 12 49.2 nmolˑl^− 1^ (median) 19.8 ngˑml^− 1^ (median) 31.6–65.0 nmolˑl^− 1^ 12.7–26.1 ngˑml^− 1^	*n* = 21 62.9 nmolˑl^− 1^ (median) 25.3 ngˑml^− 1^ (median) 55.7–67.7 nmolˑl^− 1^ 22.4–27.2 ngˑml^− 1^
Flueck et al. 2016a	Total year	*n* = 108 60.4 ± 23.2 nmolˑl^− 1^ 24.3 ± 9.3 ngˑml^− 1^ 13–119 nmolˑl^− 1^ 5–48 ngˑml^− 1^	*n* = 55 59.2 ± 24.1 nmolˑl^− 1^ 23.8 ± 9.7 ngˑml^− 1^ 16–118 nmolˑl^− 1^ 6–47 ngˑml^− 1^	*n* = 47 53.9 ± 24.7 nmolˑl^− 1^ 21.6 ± 9.9 ngˑml^− 1^ 13–117 nmolˑl^− 1^ 5–47 ngˑml^− 1^	*n* = 116 62.5 ± 22.6 nmolˑl^− 1^ 25.1 ± 9.1 ngˑml^− 1^ 16–119 nmolˑl^− 1^ 6–48 ngˑml^− 1^	*n* = 159 60.2 ± 23.6 nmolˑl^− 1^ 24.2 ± 9.5 ngˑml^− 1^ 13–119 nmolˑl^− 1^ 5–48 ngˑml^− 1^	-
	Summer	*n* = 53 71.1 ± 21.0 nmolˑl^− 1^ 28.6 ± 8.4 ngˑml^− 1^ 32–119 nmolˑl^− 1^ 13–48 ngˑml^− 1^	*n* = 24 65.8 ± 22.3 nmolˑl^− 1^ 26.4 ± 9.0 ngˑml^− 1^ 37–118 nmolˑl^− 1^ 15–47 ngˑml^− 1^	*n* = 29 63.1 ± 22.9 nmolˑl^− 1^ 25.3 ± 9.2 ngˑml^− 1^ 32–117 nmolˑl^− 1^ 13–47 ngˑml^− 1^	*n* = 48 73.4 ± 19.7 nmolˑl^− 1^ 29.5 ± 7.9 ngˑml^− 1^ 37–119 nmolˑl^− 1^ 15–48 ngˑml^− 1^	*n* = 77 69.5 ± 21.4 nmolˑl^− 1^ 27.9 ± 8.6 ngˑml^− 1^ 32–119 nmolˑl^− 1^ 13–48 ngˑml^− 1^	-
	Winter	*n* = 55 50.0 ± 20.5 nmolˑl^− 1^ 20.1 ± 8.2 ngˑml^− 1^ 13–96 nmolˑl^− 1^ 5–39 ngˑml^− 1^	*n* = 31 54.1 ± 24.5 nmolˑl^− 1^ 21.7 ± 9.8 ngˑml^− 1^ 16–109 nmolˑl^− 1^ 6–44 ngˑml^− 1^	*n* = 18 39.1 ± 20.1 nmolˑl^− 1^ 15.7 ± 8.1 ngˑml^− 1^ 13–82 nmolˑl^− 1^ 5–33 ngˑml^− 1^	*n* = 68 54.8 ± 21.3 nmolˑl^− 1^ 22.0 ± 8.6 ngˑml^− 1^ 16–109 nmolˑl^− 1^ 6–44 ngˑml^− 1^	*n* = 86 51.5 ± 21.9 nmolˑl^− 1^ 20.7 ± 8.8 ngˑml^− 1^ 13–109 nmolˑl^− 1^ 5–44 ngˑml^− 1^	-
Flueck et al. 2016b	Winter	*n* = 33 44 ± 18 nmolˑl^− 1^ 17.7 ± 7.2 ngˑml^− 1^	-	*n* = 33 44 ± 18 nmolˑl^− 1^ 17.2 ± 7.2 ngˑml^− 1^	-	*n* = 33 44 ± 18 nmolˑl^− 1^ 17.2 ± 7.2 ngˑml^− 1^	-
Pritchett et al. 2016	Autumn	*n* = 19 66.1 ± 21.2 nmolˑl^− 1^ 26.5 ± 8.5 ngˑml^− 1^	*n* = 20 73.1 ± 18.5 nmolˑl^− 1^ 29.4 ± 7.4 ngˑml^− 1^	*n* = 15 66.3 ± 21.5 nmolˑl^− 1^ 26.6 ± 8.6 ngˑml^− 1^	*n* = 24 74.8 ± 16.2 nmolˑl^− 1^ 30.0 ± 6.5 ngˑml^− 1^	*n* = 39 70.0 ± 19.6 nmolˑl^− 1^ 28.1 ± 2.9 ngˑml^− 1^	-
	Winter	*n* = 19 70.1 ± 28 nmolˑl^− 1^ 28.2 ± 11.2 ngˑml^− 1^	*n* = 20 65.2 ± 23.2 nmolˑl^− 1^ 26.2 ± 9.3 ngˑml^− 1^	*n* = 15 66.1 ± 28.5 nmolˑl^− 1^ 26.2 ± 11.4 ngˑml^− 1^	*n* = 24 69.8 ± 21.5 nmolˑl^− 1^ 28.0 ± 8.6 ngˑml^− 1^	*n* = 39 68.3 ± 19.2 nmolˑl^− 1^ 27.4 ± 7.7 ngˑml^− 1^	-
Pritchett et al. 2017	Winter	-	-	-	-	*n* = 35 66.3 ± 24.3 nmolˑl^− 1^ 26.6 ± 9.8 ngˑml^− 1^	-
Baranauskas et al. 2020	Summer	*-*	*n* = 14 60.0 ± 16.4 nmolˑl^− 1^ 24.1 ± 6.6 ngˑml^− 1^	*n* = 14 60.0 ± 16.4 nmolˑl^− 1^ 24.1 ± 6.6 ngˑml^− 1^	-	-	*n* = 14 60.0 ± 16.4 nmolˑl^− 1^ 24.1 ± 6.6 ngˑml^− 1^
Langley et al. 2021	Winter	*n* = 22 46.6 ± 18.2 nmolˑl^− 1^ 18.7 ± 7.3 ngˑml^− 1^	-	-	*n* = 22 46.6 ± 18.2 nmolˑl^− 1^ 18.7 ± 7.3 ngˑml^− 1^	-	*n* = 22 46.6 ± 18.2 nmolˑl^− 1^ 18.7 ± 7.3 ngˑml^− 1^
Langley et al. 2023	Winter	*n* = 15 40.1 ± 15.7 nmolˑl^− 1^ 16.1 ± 6.3 ngˑml^− 1^	-	-	*n* = 15 40.1 ± 15.69 nmolˑl^− 1^ 16.1 ± 6.3 ngˑml^− 1^	-	*n* = 15 40.1 ± 15.69 nmolˑl^− 1^ 16.1 ± 6.3 ngˑml^− 1^
	Summer	*n* = 15 76.7 ± 31.1 nmolˑl^− 1^ 30.8 ± 12.5 ngˑml^− 1^	-	-	*n* = 15 76.7 ± 31.1 nmolˑl^− 1^ 30.8 ± 12.5 ngˑml^− 1^	-	*n* = 15 76.7 ± 31.1 nmolˑl^− 1^ 30.8 ± 12.5 ngˑml^− 1^
Hertig-Godeschalk et al. 2023	March – October (season not specified)	-	-	-	-	*n* = 14 72 ± 17 nmolˑl^− 1^ 28.9 ± 6.8 ngˑml^− 1^	-
Steffen et al. 2024	Winter	-	-	-	-	*-*	-
	Spring	-	-	-	-	*-*	-
	Summer	-	-	-	-	*-*	-
	Autumn	-	-	-	-	*-*	-

When examining vitamin D status, in the context of training environment, of the Para-Athletes, one study reported sufficient vitamin D in outdoor Para-Athletes in the summer months [49] where a second study found outdoor Para-Athletes in the summer months to be insufficient (50–75 nmolˑl^− 1^) [41]. Two studies reported insufficient 25(OH)D levels in indoor Para-Athletes in the summer months [45, 50] and one study reported indoor athletes to be deficient in 25(OH)D (< 50 nmolˑl^− 1^) [50]. Of the studies where sampling took place during the winter months, two studies reported insufficient 25(OH)D levels (50–75 nmolˑl^− 1^) in outdoor Para-Athletes [46, 50] and two studies reported deficiency in outdoor Para-Athletes [34, 49]. Indoor Para-Athletes, in the winter, were reported to be insufficient (50–75 nmolˑl^− 1^) by one study [46] and a further study reported indoor Para-Athletes to be deficient (< 50 nmolˑl^− 1^) [50]. Pritchett et al. [46] reported vitamin D status in autumn in both indoor and outdoor Para-Athletes, of which both indoor and outdoor athletes were insufficient on average (See Fig. 2B). Two of the included studies investigated the difference between indoor and outdoor Para-Athletes [46, 50]. Flueck et al. [50] reported that outdoor Para-Athletes had significantly higher (*p* < 0.05) 25(OH)D levels compared to indoor Para-Athletes in summer, winter, and the combined seasonal averages for a whole year. Whereas Pritchett et al. [46] reported no significant difference in 25(OH)D among indoor and outdoor Para-Athletes in the autumn (*p* = 0.19) or winter months (*p* = 0.75).

### Ambulation Levels

**Fig. 3:**
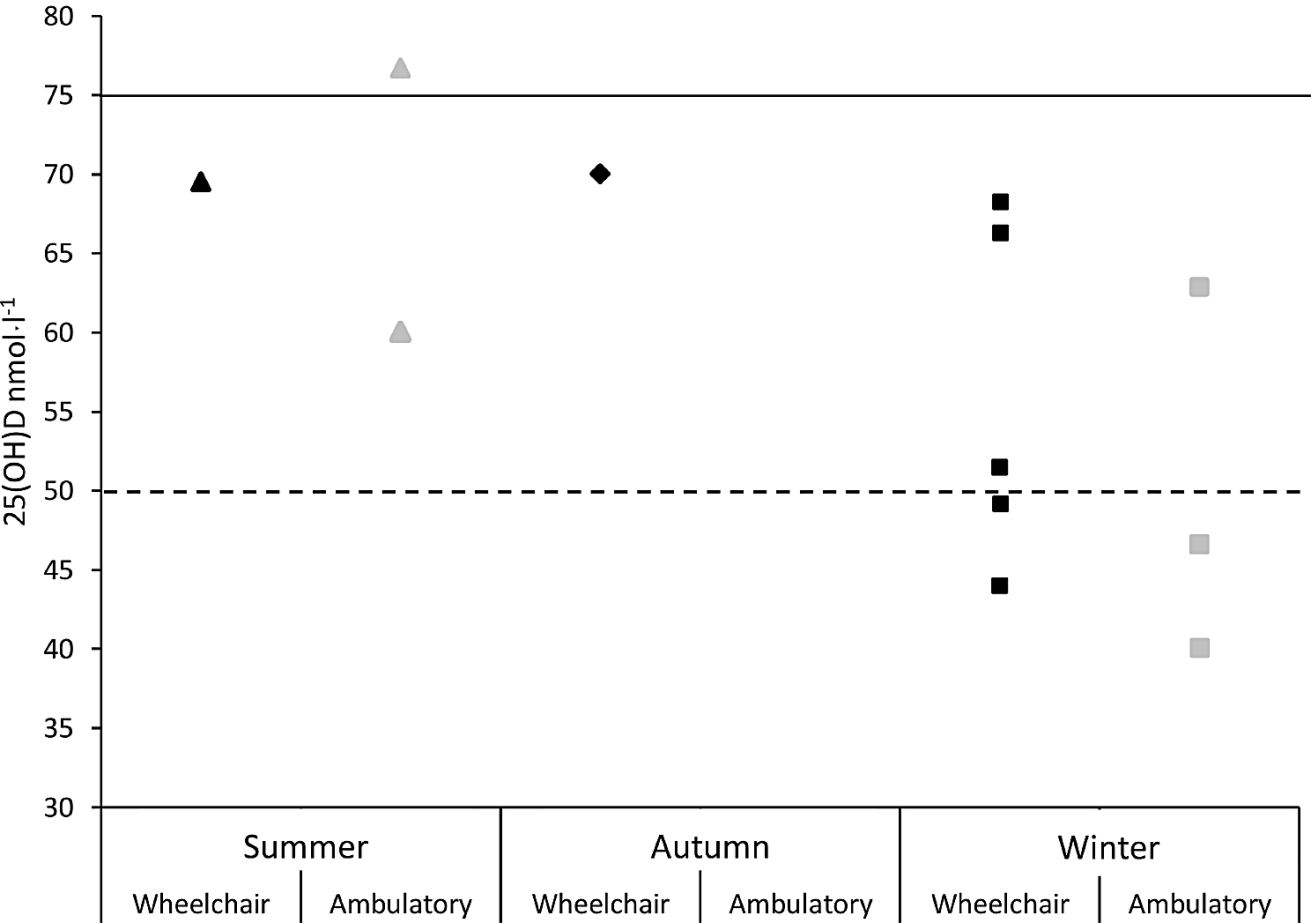
Illustrates the reported 25(OH)D levels in ambulatory (grey markers) vs. wheelchair-based Para-Athletes (black markers) over each season where data was reported. Summer denoted by triangles, autumn denoted by diamond and winter denoted by squares, spring 25(OH)D was not reported by any study. Solid line denotes threshold for vitamin D insufficiency (50–75 nmolˑl^− 1^) and dashed line denotes threshold for vitamin D deficiency (< 50 nmolˑl^− 1^). One study reporting WC Para-Athletes did not distinguish the season measured [51]

Nine of the ten studies distinguished the status of ambulation of their sampled participants i.e., ambulatory or WC based Para-Athletes. Five out of the nine studies measured WC Para-Athletes only (*n* = 181), three studies measured ambulatory Para-Athletes only (*n* = 54) and one measured both ambulatory and WC based Para-Athletes (WC *n* = 12 and ambulatory *n* = 21). Totalling *n* = 193 WC Para-Athletes and *n* = 75 ambulatory Para-Athletes (see Table 2 for information on specific impairment types). Steffen et al. [44] did not identify the ambulation levels of their Para-Athletes. In the summer months, one study reported sufficient 25(OH)D levels [49] and one study reported insufficient levels of 25(OH)D in ambulatory Para-Athletes [45]. The one study including WC Para-Athletes that measured 25(OH)D in the summer, reported insufficiency [50]. Autumn values were only sampled by one study and highlighted 25(OH)D insufficiency in WC Para-Athletes [46]. During the winter one study reported 25(OH)D levels to be insufficient [43] and two studies reported 25(OH)D levels to be deficient in ambulatory Para-Athletes [34, 49]. Whereas five studies measured 25(OH)D in the winter with WC Para-Athletes, in which three studies reported insufficiency [42, 46, 50] and two studies reported deficiency [41, 43]. One study reported insufficiency in their WC Para-Athletes but did not distinguish between the seasons/months measured (March-October) and therefore it cannot be delineated as to the seasonality of this measurement [51]. Only one study directly compared 25(OH)D in WC and ambulatory Para-Athletes [43]. Magee et al. [43] reported that WC Para-Athletes had a significantly lower 25(OH)D levels (*p* < 0.035) compared to more ambulatory Para-Athletes. None of the other included studies took samples from both WC and ambulatory Para-Athletes, which meant no further direct comparisons have been made within previous literature (See Fig. 3).

## Discussion

To the authors’ knowledge this systematic review, is the first review to examine previous studies which have measured and reported un-supplemented vitamin D levels in elite Para-Athletes with the aim to identify the prevalence of vitamin D insufficiency and deficiency (low vitamin D) in these elite Para-Athletes and the potential risk factors that may account towards reductions in vitamin D. This systematic review identified *n* = 355 Para-Athletes from the ten included studies who provide *n* = 548 samples, based upon the 25(OH)D insufficiency and deficiency thresholds set by each individual study *n* = 372 (68.1%) samples taken across different time points in the year from the Para-Athletes were insufficient. Overall, this systematic review presents a new body of evidence that demonstrates that elite Para-Athletes living at latitudes > 37^o^N are at high risk of vitamin D insufficiency and deficiency throughout the year.

When interpreting the findings of this review and thus the findings of the included studies, it is important to note that eight of the ten studies used the threshold for vitamin D sufficiency as > 75 nmolˑl^− 1^ (or 30 ngˑml^− 1^) which was set by The Endocrine Society [2]. However, Magee et al. [43] used a lower threshold for insufficiency of < 50 nmolˑl^− 1^ compared to the other included studies which may explain their higher levels of vitamin D sufficiency. The median value for the Para-Athletes in Magee et al. [43] was 57.9 nmolˑl^− 1^ which using the more common thresholds of > 75 nmolˑl^− 1^ for sufficiency would likely increase the prevalence of insufficiency in their sampled Para-Athletes. Using a lower threshold may reduce the urgency for treatment such as supplementation of vitamin D, which may lead to implications on performance, reductions in muscle function [52] and increase risk of fracture [53]. Initially, Pritchett et al. [46] set a sufficiency threshold of > 80 nmolˑl^− 1^ in their study published in 2016, however in the follow up article published in 2017 [42] they chose to set the threshold at > 75 nmolˑl^− 1^ in line with the majority of the included studies to align with the thresholds set by The Endocrine Society [2].

The ability to directly interpret vitamin D status was limited, due to various factors including the differences in thresholds for insufficiency and sufficiency set by the included studies. Furthermore, one of the included studies misreported their 25(OH)D units as mmolˑl^− 1^ when the values aligned with ngˑml^− 1^ [41]. To ensure interpretation was done correctly the first author was contacted and it was confirmed that the units had been misreported as mmolˑl^− 1^ rather than the intended units of ngˑml^− 1^. This however did not impact the interpretation and reporting of the prevalence of vitamin D insufficiency or deficiency in their study and therefore interpretation is correct in this systematic review [45]. Another factor that may have impacted the interpretation and comparison of 25(OH)D prevalence was the use of different methods of 25(OH)D analysis across the majority of included studies, of which none used the gold standard liquid chromatography with tandem mass spectrometry (LC- MS/MS) [54]. Therefore, the accuracy of the reported 25(OH)D values may have been compromised due to use of techniques such as ELISA and ECLIA that have been shown to typically over and underestimate 25(OH)D levels due to cross-reactivity [54, 55].

This current review found that the low vitamin D risk is higher in elite Para-Athletes when compared to previous findings of vitamin D status in athletes without impairment. For example, Harju et al. [56] reported an average 25(OH)D of 66.4 ± 30.4 nmolˑl^− 1^ among *n* = 3725 elite athletes compared to 62.6 ± 22.9 nmolˑl^− 1^ in the elite Para-Athletes sampled within this review. However, due to the insufficiency cut-off being set at < 50 nmolˑl^− 1^ in the Harju et al. [56] review compared to the threshold of < 75 nmolˑl^− 1^ used in this current review any prevalence data cannot be directly compared. The inconsistent use of 25(OH)D threshold cut-offs creates a challenge for the comparison of 25(OH)D results. For example, when comparing a similar systematic review that sampled athletes, Farrokhyar et al. [57] identified a low vitamin D prevalence of 56% compared to this review of 68.1% in elite Para-Athletes. However, Farrokhyar et al. [57] used a higher vitamin D insufficiency threshold cut-off of < 80 nmolˑl^− 1^ compared to < 75 nmolˑl^− 1^ which may highlight a decreased prevalence of low vitamin D in athletes compared to if they had used the lower threshold and consequently, differences in low vitamin D prevalence might be even greater in elite Para-Athletes compared to other athletic populations if a consistent threshold cut-offs had been used.

Dietary vitamin D intake was found to be low in all included studies that measured and reported this outcome variable [34, 42, 43, 45, 46] when compared to the IOM’s recommendations of 400-600IU/d. Within the studies that measured dietary vitamin D they all reported that there was no relationship between dietary vitamin D and 25(OH)D levels. Low dietary vitamin D intake is not restricted to elite Para-Athletes but common and comparable to healthy adult populations [48] and athletes [58]. Spiro and Buttriss [48] found that healthy male and female adults living in ten different European countries (latitudes ranging between 38–59°N) had poor dietary intake below the RDI (range 1.6–7.1 µg/d (64–284 IU/d)) except for adults living in Norway who had a dietary intake of between 10.1 and 10.9 µg/day (404–436 IU/d) thus meeting the RDI. Similarly, García and Guisado [58] found that a group of 21 elite male basketballers living in Barcelona (41°N) had an average vitamin D intake of 139 ± 79 (range 16–282) IU/d [58]. García and Guisado [58] also reported serum 25(OH)D levels positively correlated with dietary intake of vitamin D (*r* = 0.65; *p <* 0.001). Whereas in the studies involving Para-Athletes in the present review, no association was reported between 25(OH)D and dietary vitamin D. It is possible that a lack of association between dietary intake and 25(OH)D in Para-Athletes in the current studies is attributable to all studies reporting deficient dietary intakes, with very high prevalence of 25(OH)D insufficiency; and as a result, there was a lack of outcome variability from which an association could be found [59]. Therefore, it is of importance that Para-Athletes should understand major dietary vitamin D sources and regularly track their dietary vitamin D intakes with the aim to meet the RDI’s to help achieve sufficient 25(OH)D levels.

When considering the influence of sex on vitamin D status, there were no significant differences reported by any of the included studies that compared vitamin D status between male and female elite Para-Athletes. This finding aligns with a previous systematic review and meta-analysis of eight studies that investigated sex differences in healthy elite male and female athletes, which concluded that after within-study comparisons that there was no significant sex difference (RR = 1.0; 95% CI 0.79–1.26) [56]. However, due to the heterogeneous sample within this review, confounded by the few studies, small sample sizes, low female Para-Athlete representation (*n* = 108/355 or 30.4%), diverse sports and varying impairment types, it is therefore unlikely that the 25(OH)D levels presented are a true reflection of the level of insufficiency and deficiency of female Para-Athletes. It should be noted that the current knowledge of sex differences between healthy male and female populations (adults, children, etc.) is still unanswered, where there is currently no known research that identifies exact mechanisms that are able to categorically explain sex differences on vitamin D status and therefore cannot be truly answered in athletic populations [60].

The latitudes of all included studies in this review were > 37°N, whereby it was expected and shown that the prevalence of deficiency and insufficiency was greater in the winter months compared to the autumn and summer, due to seasonal variations making UVb exposure extremely low or even negligible during the winter, at this latitude and above [61]. This seasonal variation in vitamin D found within this review is consistent with previous research published in athletes, were for example Morton et al. [62] showed seasonal decreases of serum 25(OH)D from optimal levels in the summer (104.4 ± 21.1 nmolˑl^− 1^) to insufficient in the winter (51.0 ± 19.0 nmolˑl^− 1^) in 20 professional footballers living in the UK at 53°N. However, unlike athletes, the included studies and thus elite Para-Athletes sampled within this review demonstrated that the prevalence of insufficiency remained high in the summer months apart from one group of ambulatory Para-Athletes [49]. The one study which reported sufficient 25(OH)D in the summer months, sampled outdoor Para-Athletes residing at 53°N, which was one of the highest latitudes included in this review [49]. Despite these Para-Athletes having less available sun exposure during the summer at 53°N compared to the studies with Para-Athletes residing at lower latitudes included in this review, it is possible that they were sufficient due to other lifestyle factors including playing an outdoor sport and being more ambulatory. One study that measured seasonal variations [46] reported that there was no significant seasonal variation in their sampled indoor and outdoor Para-Athletes. This lack of seasonal variation contrasts with the vast literature which typically supports the significant changes in vitamin D from seasonal variation shown in previous research that concerns both Para-Athletes [49] and athletes without impairments [62]. The lack of seasonal variation outlined by Pritchett et al. [46] is likely due to 25(OH)D being measured in the autumn followed by the winter, which likely captured vitamin D at its nadir but not its peak, which is typically during the summer months [63]. Therefore, when comparing 25(OH)D from autumn to winter, Pritchett et al. [46] may have not seen a great enough change in vitamin D for it to be significant due to sampling in consecutive seasons. Nonetheless, all articles in this review suggest that many Para-Athletes are at risk of vitamin D insufficiency and deficiency all year round.

This systematic review identified that based upon the 25(OH)D averages in each of the included studies that there were no vitamin D sufficient indoor Para-Athletes and that there was just one study that reported sufficient levels of vitamin D in outdoor Para-Athletes in the summer. This review shows similar results when compared to previous research, which suggests that indoor athletes have lower vitamin D levels compared to outdoor athletes. Flueck et al. [50] reported consistent results when compared with previous studies that investigated training environment differences in athletes, concluding that indoor Para-Athletes were at higher risk of vitamin insufficiency and deficiency. The influence of training environment is echoed in a study investigating seasonal variations in 30 (15 indoor, 15 outdoor) female college athletes, which found outdoor athletes to have 25(OH)D levels of 13 ngˑml^− 1^ (32.37 nmolˑl^− 1^) more in the winter (March) and 7 ngˑml^− 1^ (17.43 nmolˑl^− 1^) more in the summer (September) compared to indoor athletes [64]. However, one of the retrieved articles, Pritchett et al. [46] reported no differences between indoor and outdoor athletes, this may be due to measures being completed in autumn and winter months, which would mean that any outdoor UVb exposure would likely be negligible, or too low to have a significant impact on serum 25(OH)D levels. Therefore, these outdoor Para-Athletes would likely experience similar vitamin D exposure to their indoor counterparts [61]. Importantly, the findings from this review would encourage for indoor Para-Athletes to take greater pre-cautions all year round, such as aiming for more safe sun exposure and/or supplementation, even during the summer months, when trying to avoid vitamin D insufficiency and deficiency.

The level of ambulation of the sampled elite Para-Athletes may influence the training environment and thus time spent outdoors which has been discussed above and shown to see a reduction in 25(OH)D. Within the included studies Magee et al. [43], was the only study to directly compare WC-bound Para-Athletes to more ambulatory Para-Athletes and reported significantly lower 25(OH)D levels among WC-bound Para-Athletes. As sun exposure is related to outdoor time [65] and outdoor time has been shown to reduce with increased levels of sedentary behaviour, it could be suggested that individuals who have less physical function may be at higher risk of lower levels of 25(OH)D [7]. This suggestion can be supported by Nooijen et al. [7] who reported that compared to able-bodied controls, ambulatory individuals with cerebral palsy were more sedentary (80 min) and performed less physical activity (48 min). Therefore, it is of importance for Para-Athletes who are WC-bound or have reduced physical function to acknowledge and modify their lifestyle factors that can improve their vitamin D levels, such as safe sun exposure in the summer months, vitamin D supplementation and improved diet.

### Limitations

Due to the lack of homogeneity between the included studies, which is commonly seen in studies investigating Para-Athletes, due to varying impairment types and severity, we were unable to further synthesise and analyse the data from the included studies via meta-analysis. Therefore, while it was possible to ascertain a direction of the possible detected relationships, we were not able to quantify the overall effect size for each outcome. Of the ten included studies, there were publications which were secondary studies performed by the same authors, who likely collected or reused previous measurements of 25(OH)D from some of the same participants. This would therefore reduce the overall population size of unique participants that 25(OH)D is reflected in. Nevertheless, this review is the first to identify the prevalence of low vitamin D in elite Para-Athletes around the world with varying impairments.

### Future Recommendations

Recommendations for future research are; (1) Standardised thresholds for vitamin D status: To facilitate direct comparisons, researchers should adopt standardised thresholds for assessing vitamin D levels such as those recommended by The Endocrine Society; (2) Exploring the impact of vitamin D on female Para-Athletes: Given the underrepresentation of female populations in existing research and as identified in this systematic review, further investigation into the effects of vitamin D on female Para-Athletes is crucial; (3) Expanding the research in Para-Sports and different impairment types: This broader exploration into Para-Athletes will contribute the growth of the existing literature in this field. It is also recommended that practitioners should; (1) Proactively monitor their Para-Athletes vitamin D: To highlight if there is need for intervention such as, supplementation to avoid decrements in musculoskeletal function and health; (2) Provide education on the sources and importance of vitamin D: Including dietary vitamin D rich sources and methods on how to increase circulating vitamin D through safe sun exposure.

## Conclusion

Ten studies were included within this systematic review, where a high prevalence of low vitamin D (57.1% - summer and 74.1% - winter) was identified in elite Para-Athletes, living at latitudes above 37°N. The included studies identified several risk factors which likely influence low vitamin D in Para-Athletes, including lower ambulation levels, training environment (indoor vs. outdoor), poor dietary intake and access to vitamin D rich sources and seasonal variations in sun exposure. This systematic review highlighted that vitamin D insufficiency rates were high in the summer months, demonstrating that low vitamin D may be an issue for Para-Athletes throughout the entire year. Therefore, the authors recommend the need for interventions such as improved diet, increased outdoor exposure and supplementation of vitamin D throughout the year.

## Data Availability

All extracted data is included within this manuscript.

## Footnotes

1 Please note - Vitamin D deficiency is defined by most experts and The Endocrine Society as a total 25-hydroxyvitamin D level < 50 nmolˑl^−1^ (< 20 ngˑml^−1^). Vitamin D insufficiency is defined as a level of between 50 and 75 nmolˑl^− 1^ (20–30 ngˑml^− 1^), and > 75 nmolˑl^− 1^ (> 30 ngˑml^− 1^) or greater indicates sufficient levels. Moreso, an ‘optimal’ threshold is described by most as > 100 nmolˑl^− 1^ (>40 ngˑml^− 1^). These will be the thresholds used by the authors in this review. However due to the nature of this review some published literature identified may use different thresholds to define deficiency which will be identified throughout.

## Abbreviations

25(OH)D: 25 hydroxyvitamin D
CP: Cerebral Palsy
ECLIA: Electrochemiluminescence immunoassay
ELISA: Enzyme linked immunosorbent assay
F: Female
IOM: Institute of Medicine
IPAQ: International Physical Activity Questionnaire
JBI: Joanna Briggs Institute
M: Male
NS: Not specified
PA: Physical activity
PECO: Population, Exposure, Comparison, Outcome
PRISMA: Preferred Reporting Items for Systematic reviews and Meta-Analyses
PROSPERO: Prospective Register of Systematic Reviews
RDI: Recommended daily intake
SB: Spina Bifida
SCI: Spinal cord injury
TDC: Typically developed controls
UK: United Kingdom
USA: United Stated of America
UV b: Ultraviolet beta
WC: Wheelchair

## References

[1] Holick MF, Chen TC. Vitamin D deficiency: a worldwide problem with health consequences. Am J Clin Nutr. 2008;87(4):S1080–6. 10.1093/ajcn/87.4.1080S 18400738

[2] Amrein K, et al. Vitamin D deficiency 2.0: an update on the current status worldwide. Eur J Clin Nutr. 2020;74(11):1498–513. 31959942 10.1038/s41430-020-0558-y PMC7091696

[3] Dominguez LJ, et al. Vitamin D sources, metabolism, and deficiency: available compounds and guidelines for its treatment. Metabolites. 2021;11(4):255. 33924215 10.3390/metabo11040255 PMC8074587

[4] Chen TC, et al. Factors that influence the cutaneous synthesis and dietary sources of vitamin D. Arch Biochem Biophys. 2007;460(2):213–7. 17254541 10.1016/j.abb.2006.12.017 PMC2698590

[5] Maxwell J. Seasonal variation in vitamin D. Proc Nutr Soc. 1994;53(3):533–43. 7886053 10.1079/PNS19940063

[6] Davie M, et al. Vitamin D from skin: contribution to vitamin D status compared with oral vitamin D in normal and anticonvulsant-treated subjects. Clin Sci (London England: 1979). 1982;63(5):461–72. 10.1042/cs0630461 6288317

[7] Nooijen CF, et al. Inactive and sedentary lifestyles amongst ambulatory adolescents and young adults with cerebral palsy. J Neuroeng Rehabil. 2014;11(1):1–8. 24708559 10.1186/1743-0003-11-49 PMC4002542

[8] DeLuca HF. Overview of general physiologic features and functions of vitamin D. Am J Clin Nutr. 2004;80(6):S1689–96. 10.1093/ajcn/80.6.1689S 15585789

[9] Goltzman D. Functions of vitamin D in bone. Histochem Cell Biol. 2018;149(4):305–12. 29435763 10.1007/s00418-018-1648-y

[10] Bischoff-Ferrari HA, Orav EJ, Dawson-Hughes B. Effect of cholecalciferol plus calcium on falling in ambulatory older men and women - A 3-year randomized controlled trial. Arch Intern Med. 2006;166(4):424–30. 16505262 10.1001/archinte.166.4.424

[11] Bischoff HA, et al. Effects of vitamin D and calcium supplementation on falls: a randomized controlled trial. J Bone Miner Res. 2003;18(2):343–51. 12568412 10.1359/jbmr.2003.18.2.343

[12] Bischoff-Ferrari HA, et al. Fracture prevention with vitamin D supplementation: a meta-analysis of randomized controlled trials. JAMA. 2005;293(18):2257–64. 15886381 10.1001/jama.293.18.2257

[13] Tuohimaa P. Vitamin D and aging. J Steroid Biochem Mol Biol. 2009;114(1–2):78–84. 19444937 10.1016/j.jsbmb.2008.12.020

[14] Schwalfenberg GK. A review of the critical role of vitamin D in the functioning of the immune system and the clinical implications of vitamin D deficiency. Volume 55. Molecular nutrition & food research; 2011. pp. 96–108. 1. 10.1002/mnfr.201000174 20824663

[15] Tanner SB, Harwell SA. More than healthy bones: a review of vitamin D in muscle health. Therapeutic Adv Musculoskelet Disease. 2015;7(4):152–9. 10.1177/1759720X15588521 PMC4530385 26288665

[16] Montenegro KR, et al. Vitamin D supplementation does not impact resting metabolic rate, body composition and strength in vitamin D sufficient physically active adults. Nutrients. 2020;12(10):15. 10.3390/nu12103111 PMC7601703 33053823

[17] Webster J, et al. Nutritional strategies to optimise musculoskeletal health for fall and fracture prevention: looking beyond calcium, vitamin D and protein. Bone Rep. 2023;101684:p. 10.1016/j.bonr.2023.101684 PMC10757289 38163013

[18] Tomlinson PB, Joseph C, Angioi M. Effects of vitamin D supplementation on upper and lower body muscle strength levels in healthy individuals. A systematic review with meta-analysis. J Sci Med Sport. 2015;18(5):575–80. 25156880 10.1016/j.jsams.2014.07.022

[19] Larson-Meyer DE, Willis KS. Vitamin D and athletes. Curr Sports Med Rep. 2010;9(4):220–6. 20622540 10.1249/JSR.0b013e3181e7dd45

[20] Close GL, et al. The effects of vitamin D-3 supplementation on serum total 25 OH D concentration and physical performance: a randomised dose-response study. Br J Sports Med. 2013;47(11):692–6. 23410885 10.1136/bjsports-2012-091735

[21] Owens D, et al. Efficacy of high-dose vitamin D supplements for Elite athletes. Med Sci Sports Exerc. 2017;49(2):349–56. 27741217 10.1249/MSS.0000000000001105

[22] Wilson-Barnes S, et al. Effects of vitamin D on health outcomes and sporting performance: implications for elite and recreational athletes. Nutr Bull. 2020;45(1):11–24. 10.1111/nbu.12413

[23] Angeline ME, et al. The effects of vitamin D deficiency in athletes. Am J Sports Med. 2013;41(2):461–4. 23371942 10.1177/0363546513475787

[24] Koundourakis NE, et al. Discrepancy between exercise performance, body composition, and sex steroid response after a six-week detraining period in professional soccer players. PLoS ONE. 2014;9(2):e87803. 24586293 10.1371/journal.pone.0087803 PMC3929557

[25] Koundourakis NE, et al. Muscular effects of vitamin D in young athletes and non-athletes and in the elderly. Hormones. 2016;15(4):471–88. 28222403 10.14310/horm.2002.1705

[26] Larson-Meyer D. The importance of vitamin D for athletes. Sports Sci Exch. 2015;28:1–8.

[27] Constantini NW, et al. High prevalence of vitamin D insufficiency in athletes and dancers. Clin J Sport Med. 2010;20(5):368–71. 20818195 10.1097/JSM.0b013e3181f207f2

[28] Close GL, et al. Assessment of vitamin D concentration in non-supplemented professional athletes and healthy adults during the winter months in the UK: implications for skeletal muscle function. J Sports Sci. 2013;31(4):344–53. 23083379 10.1080/02640414.2012.733822

[29] Lovell G. Vitamin D status of females in an elite gymnastics program. Clin J Sport Med. 2008;18(2):159–61. 18332692 10.1097/JSM.0b013e3181650eee

[30] Wyon MA, et al. The influence of winter vitamin D supplementation on muscle function and injury occurrence in elite ballet dancers: a controlled study. J Sci Med Sport. 2014;17(1):8–12. 23619160 10.1016/j.jsams.2013.03.007

[31] Dahlquist DT, Dieter BP, Koehle MS. Plausible ergogenic effects of vitamin D on athletic performance and recovery. J Int Soc Sports Nutr. 2015;12(1):33. 26288575 10.1186/s12970-015-0093-8 PMC4539891

[32] Książek A, Zagrodna A, Słowińska-Lisowska M. Vitamin D, skeletal muscle function and athletic performance in athletes—A narrative review. Nutrients. 2019;11(8):1800. 31382666 10.3390/nu11081800 PMC6722905

[33] Wyon MA, et al. Effect of vitamin D on muscle function and Injury in Elite Adolescent dancers: a Randomized double-blind study. Int J Sports Physiol Perform. 2019;14(1):55–9. 29893596 10.1123/ijspp.2018-0084

[34] Langley CK et al. Musculoskeletal Health in active ambulatory men with cerebral palsy and the impact of Vitamin D. Nutrients, 2021. 13(7). 10.3390/nu13072481 PMC8308596 34371988

[35] Hussain AW, et al. Muscle size, activation, and coactivation in adults with cerebral palsy. Muscle Nerve. 2014;49(1):76–83. 23558961 10.1002/mus.23866

[36] Lee MR, et al. Independently ambulatory children with spina bifida experience near-typical knee and ankle joint moments and forces during walking. Gait Posture. 2023;99:1–8. 36283301 10.1016/j.gaitpost.2022.10.010 PMC9772073

[37] Rolvien T, Amling M. *Disuse osteoporosis: clinical and mechanistic insights* Calcified tissue international, 2022: pp. 1–13. 10.1007/s00223-021-00836-1 PMC9013332 33738515

[38] Zarandi HP, et al. Comparing the Mental Health of the athletic and non-athletic physically-disabled people. Iran J Health Phys Activity. 2011;2(1):6–10.

[39] Cavedon V, et al. Body composition and bone mineral density in athletes with a physical impairment. PeerJ. 2021;9:e11296. 34026349 10.7717/peerj.11296 PMC8117930

[40] Page MJ, et al. The PRISMA 2020 statement: an updated guideline for reporting systematic reviews. Int J Surg. 2021;88:105906. 33789826 10.1016/j.ijsu.2021.105906

[41] Flueck JL, Schlaepfer M, Perret C. Effect of 12-Week vitamin D supplementation on 25 OH D Status and performance in athletes with a spinal cord Injury. Nutrients. 2016;8(10):13. 27669288 10.3390/nu8100586 PMC5083975

[42] Pritchett KL, et al. Effect of vitamin D supplementation on 25(OH)D Status in Elite athletes with spinal cord Injury. Med Sci Sports Exerc. 2017;49(5):678–678. 10.1249/01.mss.0000518790.98957.ea 29757043

[43] Magee PJ, et al. Vitamin D status and supplementation in elite Irish athletes. Int J Sport Nutr Exerc Metab. 2013;23(5):441–8. 23535936 10.1123/ijsnem.23.5.441

[44] Steffen K et al. Comprehensive periodic health evaluations of 454 Norwegian paralympic and olympic athletes over 8 years: what did we learn? Br J Sports Med, 2024. 10.1136/bjsports-2023-107942 PMC11287577 38744502

[45] Baranauskas M, et al. Cardiorespiratory Fitness and Diet Quality Profile of the Lithuanian Team of Deaf Women’s basketball players. Int J Environ Res Public Health. 2020;17(18):17. 10.3390/ijerph17186749 PMC7560087 32947980

[46] Pritchett K et al. 25(OH)D Status of Elite athletes with Spinal Cord Injury Relative to lifestyle factors. Nutrients, 2016. 8(6). 10.3390/nu8060374 PMC4924215 27322316

[47] Holick MF, et al. Evaluation, treatment, and prevention of vitamin D deficiency: an endocrine Society clinical practice guideline. J Clin Endocrinol Metabolism. 2011;96(7):1911–30. 10.1210/jc.2011-0385 21646368

[48] Spiro A, Buttriss J. Vitamin D: an overview of vitamin D status and intake in E urope. Nutr Bull. 2014;39(4):322–50. 25635171 10.1111/nbu.12108 PMC4288313

[49] Langley CK, et al. Seasonal variations in vitamin D do not change the musculoskeletal health of physically active ambulatory men with cerebral palsy: a longitudinal cross-sectional comparison study. Nutr Res. 2023;111:24–33. 36812881 10.1016/j.nutres.2022.11.005

[50] Flueck JL, et al. Vitamin D deficiency in Swiss elite wheelchair athletes. Spinal Cord. 2016;54(11):991–5. 26976532 10.1038/sc.2016.33

[51] Hertig-Godeschalk A, et al. Energy availability and nutritional intake during different training phases of wheelchair athletes. Nutrients. 2023;15(11):2578. 37299541 10.3390/nu15112578 PMC10255248

[52] Owens DJ, Allison R, Close GL. Vitamin D and the athlete: current perspectives and new challenges. Sports Med. 2018;48:3–16. 29368183 10.1007/s40279-017-0841-9 PMC5790847

[53] Cashman KD. Vitamin D deficiency: defining, prevalence, causes, and strategies of addressing. Calcif Tissue Int. 2020;106(1):14–29. 31069443 10.1007/s00223-019-00559-4

[54] Saleh L, Mueller D, von Eckardstein A. Analytical and clinical performance of the new Fujirebio 25-OH vitamin D assay, a comparison with liquid chromatography-tandem mass spectrometry (LC-MS/MS) and three other automated assays. Clin Chem Lab Med (CCLM). 2016;54(4):617–25. 26457778 10.1515/cclm-2015-0427

[55] Fraser WD, et al. Vitamin D measurement, the debates continue, new analytes have emerged, developments have variable outcomes. Calcif Tissue Int. 2020;106:3–13. 31741016 10.1007/s00223-019-00620-2

[56] Harju T, et al. Prevalence and novel risk factors for vitamin D insufficiency in elite athletes: systematic review and meta-analysis. Eur J Nutr. 2022;61(8):3857–71. 35882673 10.1007/s00394-022-02967-z PMC9596536

[57] Farrokhyar F, et al. Prevalence of vitamin D inadequacy in athletes: a systematic-review and meta-analysis. Sports Med. 2015;45:365–78. 25277808 10.1007/s40279-014-0267-6

[58] García RB, Guisado FR. Low levels of vitamin D in professional basketball players after wintertime: relationship with dietary intake of vitamin D and calcium. Nutrición Hospitalaria. 2011;26(5):945–51. 22072336 10.1590/S0212-16112011000500004

[59] Eledum H, El-Alosey AR. Binomial-geometric mixture and its applications. Math Stat. 2022;10(6):1218–28. 10.13189/ms.2022.100608

[60] Wierzbicka A, Oczkowicz M. Sex differences in vitamin D metabolism, serum levels and action. Br J Nutr. 2022;128(11):2115–30. 35042577 10.1017/S0007114522000149

[61] Kimlin MG. Geographic location and vitamin D synthesis. Mol Aspects Med. 2008;29(6):453–61. 18786559 10.1016/j.mam.2008.08.005

[62] Morton JP, et al. Seasonal variation in vitamin D status in professional soccer players of the English Premier League. Appl Physiol Nutr Metab. 2012;37(4):798–802. 22554144 10.1139/h2012-037

[63] Cannell JJ, et al. Athletic performance and vitamin D. Sci Sports Exerc. 2009;41(5):1102–10. Medicine. 10.1249/MSS.0b013e3181930c2b 19346976

[64] Maruyama–Nagao A, Sakuraba K, Suzuki Y. Seasonal variations in vitamin D status in indoor and outdoor female athletes. Biomedical Rep. 2016;5(1):113–7. 10.3892/br.2016.671 PMC4906578 27347414

[65] Wu T, et al. Effect of vitamin D supplementation and outdoor time on the 25 (OH) D level in adolescents. Wei Sheng Yan jiu = J Hygiene Res. 2017;46(2):207–12. 29903095

